# Analysis of portal vein thrombosis prevalence and risk factors in neonates following umbilical venous catheterization: a systematic review and meta-analysis^☆^

**DOI:** 10.1016/j.jped.2026.101559

**Published:** 2026-06-15

**Authors:** Roberta Martinez Novaes, Roberta Vacari de Alcântara, Gabriel Hessel

**Affiliations:** a Programa de Saúde da Criança e do Adolescente da Faculdade de Ciências Médicas da Universidade Estadual de Campinas (UNICAMP), Campinas, SP, Brazil; b Departamento de Pediatria da Faculdade de Ciências Médicas da Universidade Estadual de Campinas (UNICAMP), Campinas, SP, Brazil

**Keywords:** Catheterization, umbilical veins, Portal vein thrombosis, Prevalence, Risk factors, Neonatal screening, Ultrasonography

## Abstract

**Objectives:**

To determine the prevalence of portal vein thrombosis (PVT) in neonates following umbilical venous catheterization (UVC), and to identify risk factors associated with PVT development in this population.

**Methods:**

Systematic review conducted in accordance with PRISMA guidelines. Prospective cohort studies assessing PVT in catheterized patients were included. Meta-analyses were performed using random-effects models for prevalence estimates and odds ratios (OR), heterogeneity assessment with Cochran's Q test and Higgins' I², and publication bias with funnel plot and Egger’s test.

**Results:**

The search identified 344 studies, of which 11 met the inclusion criteria. The sample included 1086 neonates (1052 catheterized). Abdominal ultrasound confirmed 200 cases of PVT, yielding a prevalence of 19% (range: 0% to 49%), with six studies reporting prevalence rates above 5%. Meta-analysis showed an association between PVT and blood transfusion or exchange transfusion through the umbilical catheter (*p* = 0.0024). There was a trend toward increased risk of PVT in neonates with sepsis (*p* = 0.0705), whereas catheter tip location was not associated with increased PVT prevalence, although there is a risk of bias (*p* = 0.4636).

**Conclusion:**

A wide variation in PVT prevalence was observed across the included studies. Blood transfusion through the umbilical catheter was confirmed as a risk factor, while neonatal sepsis was associated with a trend toward this outcome. These findings support the implementation of systematic abdominal ultrasound screening protocols for PVT in all neonates undergoing UVC.

## Introduction

The procedure of umbilical vessel catheterization constitutes one of the pillars of initial medical care for critically ill neonates. The “Golden Hour”, a concept adapted from emergency medicine, refers to a set of measures aimed at stabilizing the newborn, particularly the extremely premature infant, during the first postnatal hour of life [[1], [2], [3]]. To achieve this, it is essential to obtain venous access rapidly and safely [4], providing a route for the administration of medications and fluids to maintain hemodynamic stability, correct electrolyte imbalances, and establish adequate glycemic control [2].

In neonates requiring admission to a neonatal intensive care unit (NICU), central venous access often becomes mandatory due to the need for infusion of hypertonic solutions, parenteral nutrition, and inotropic agents [5,6]. The umbilical venous catheter is advantageous because of its relatively simple insertion technique [7] and its feasibility in patients with impaired peripheral perfusion, in whom insertion of a peripherally inserted central catheter (PICC) would be compromised [[8], [9], [10]].

The correlation between umbilical venous catheterization (UVC) and the occurrence of Portal Vein Thrombosis (PVT), as well as the influence of neonatal risk factors on its development, are not well defined in the medical literature [6,11]. A history of umbilical catheterization is frequently reported in retrospective studies of patients diagnosed with PVT [6,11]. However, prospective studies describe wide variability in PVT prevalence in previously catheterized patients [[12], [13], [14], [15]]. Thus, the scarcity of adequately designed studies and the variability in documented outcomes hinder the confirmation of UVC as an independent risk factor for PVT, posing an obstacle to recommending systematic screening protocols for these patients.

PVT is defined as the partial or complete obstruction of the portal venous system by an intraluminal thrombus involving the main portal vein or its intrahepatic branches, resulting in impaired portal blood flow [16]. Guidelines from the European Association for the Study of the Liver define acute PVT as typically presenting within the first three weeks of thrombus formation [16,17]. Beyond this period, persistent occlusion of the portal flow may lead to the development of periportal collateral vessels bypassing the affected segment, a process known as cavernous transformation of the portal vein [18]. Although widely adopted, these definitions remain primarily anatomical and do not adequately reflect the functional consequences of portal venous obstruction [16].

Persistent portal vein obstruction may evolve into extrahepatic portal vein obstruction (EHPVO), a clinical entity characterized by portal hypertension secondary to chronic obstruction of the extrahepatic portal vein, with or without involvement of the intrahepatic portal vein, splenic vein, or superior mesenteric vein [19]. The cavernous transformation of the portal vein, often associated with ineffective collateral circulation, contributes to increased portal venous pressure and promotes the development of portosystemic collaterals [18], thereby defining the clinical spectrum of EHPVO [20,21]. This condition has been associated with three major groups of predisposing factors: direct vascular wall injury, congenital anomalies of the portal vein, and systemic factors, including sepsis, omphalitis, other intra-abdominal infections, dehydration, abdominal surgery, and hypercoagulable states [[22], [23], [24], [25]]. Despite these associations, the etiology of EHPVO in pediatric populations remains incompletely understood, with approximately half of reported cases classified as idiopathic [20,26].

In some series, EHPVO is described as the leading cause of portal hypertension in children, particularly in low-income countries [23]. Because it is predominantly asymptomatic during the neonatal period, diagnosis is often delayed and established incidentally during abdominal ultrasound or after the occurrence of severe complications secondary to portal hypertension. These complications include splenomegaly, hypersplenism, and the development of esophageal varices, which may rupture and lead to upper gastrointestinal bleeding (UGIB), contributing to the high morbidity and mortality associated with this disease [6,11,13,24,27].

UGIB is among the most frequent clinical manifestations of EHPVO in childhood, reported in up to 79% of patients, and it is associated with high recurrence rates, averaging 2.5 episodes of gastrointestinal bleeding per patient [25,28]. A bimodal pattern of onset has also been observed: in patients with prior umbilical catheterization, the first UGIB episode tends to occur at an average age of three years, approximately five years earlier compared to idiopathic or abdominal infection-related cases of EHPVO [27].

The diagnosis of PVT and EHPVO is made by abdominal ultrasound, a low-cost, noninvasive, and highly accurate imaging modality despite its operator dependence [15,28]. When not promptly diagnosed, EHPVO carries high morbidity and mortality due to secondary complications [18,29]. Identifying PVT at an early, asymptomatic stage enables timely monitoring and reduces the incidence of major clinical consequences of portal hypertension, such as UGIB, improving patient prognosis [25,28,30].

The absence of standardized screening protocols for PVT in neonates undergoing UVC may underestimate the true prevalence of the disease in this group. Therefore, this systematic review and meta-analysis aims to prospectively analyze the prevalence of PVT in neonates previously subjected to UVC and to identify the most relevant risk factors associated with PVT development in newborns requiring umbilical catheterization at birth.

## Materials and methods

To ensure the relevance and quality of publications on the subject, study selection was performed through an extensive search of multiple electronic databases, as listed below: BVS, DeCS, MeSH, Emtree, EMBASE, PubMed, PubMed PMC, Scopus, Web of Science, and Cochrane Library. Search strategies were formulated following the steps outlined by Needleman et al. and Pollock et al. for systematic reviews [31,32]. The search criteria targeted studies evaluating PVT prevalence in patients who underwent UVC during the neonatal period. The search covered studies published between 1967 and 2024, with the last update on June 28, 2024. No restrictions were applied regarding language or publication date. The following descriptors and Boolean operators were used in the search strategy:

### Search string

((((((("Infant, Premature" OR "Premature Infant" OR "Premature Infants" OR "Preterm Infants" OR "Preterm Infant" OR "Neonatal Prematurity") OR ("Infant, Newborn" OR "Newborn Infant" OR "Newborn Infants" OR Neonate OR Neonates OR Newborns OR Newborn)) OR (Infant OR Infants)) OR ("Child, Preschool" OR "Preschool Child" OR "Preschool Children")) OR (Child OR Children)) OR (Perinatal OR Pediatric OR Paediatric)) AND (((((((Umbilical Veins[MeSH Terms]) OR ("Umbilical Veins"[Title/Abstract] OR "Umbilical Vein"[Title/Abstract])) OR ("allantoic vein"[Title/Abstract] OR "umbilical cord vein"[Title/Abstract] OR "umbilical veins"[Title/Abstract] OR "umbilicus vein"[Title/Abstract] OR "vena umbilicalis"[Title/Abstract])) AND ((((Catheterization[MeSH Terms]) OR (Catheterization[Title/Abstract] OR Catheterizations[Title/Abstract] OR Cannulation[Title/Abstract] OR Cannulations[Title/Abstract])) OR ("catheter technique"[Title/Abstract] OR catheterisation[Title/Abstract])) OR ((Catheters[MeSH Terms]) OR (Catheters[Title/Abstract] OR Catheter[Title/Abstract])))) OR ("umbilical venous catheter"[Title/Abstract])) OR ("umbilical vein catheter"[Title/Abstract])) OR ("Umbilical vein catheterization"[Title/Abstract] OR "umbilical venous catheter (UVC) "[Title/Abstract] OR "umbilical venous catheter"[Title/Abstract] OR UVC[Title/Abstract]))) AND ((((("portal vein thrombosis"[Title/Abstract] OR "portal thrombosis"[Title/Abstract] OR "portal vein thrombus"[Title/Abstract] OR pylethrombosis[Title/Abstract] OR "thrombosis, portal vein"[Title/Abstract]) OR ("extrahepatic portal venous obstruction"[Title/Abstract] OR "extrahepatic portal vein obstruction"[Title/Abstract] OR "Extrahepatic portal vein obstruction (EHPVO) "[Title/Abstract] OR "extra hepatic portal venous obstruction"[Title/Abstract])) OR ("extrahepatic portal vein thrombosis"[Title/Abstract] OR EHPVT[Title/Abstract] OR PVT[Title/Abstract])) OR ("extra-hepatic portal vein thrombosis"[Title/Abstract])) OR ((((Portal Vein[MeSH Terms]) OR ("Portal Vein"[Title/Abstract] OR "Portal Veins"[Title/Abstract])) OR ("hepatic portal vein"[Title/Abstract] OR "liver portal vein"[Title/Abstract] OR "portal tract"[Title/Abstract] OR "portal tree"[Title/Abstract] OR "portal vessel"[Title/Abstract] OR "vein, portal"[Title/Abstract] OR "vena portae"[Title/Abstract] OR "vena portae hepatis"[Title/Abstract])) AND (((Venous Thrombosis[MeSH Terms]) OR ("Venous Thrombosis"[Title/Abstract] OR "Venous Thromboses"[Title/Abstract])) OR ("vein thrombosis"[Title/Abstract]))))

### Study selection

The guidelines applied for article selection were based on the PICOS acronym (Population, Intervention, Comparison, Outcomes, and Study design) [33]:

• Population: Newborns admitted to the neonatal intensive care unit (NICU).
• Intervention: Umbilical venous catheterization.
• Comparison: Group of non-catheterized newborns.
• Primary outcome: Prevalence of PVT associated with umbilical venous catheter use.
• Secondary outcome: Identification of neonatal and procedural risk factors associated with PVT development.
• Study design: Prospective cohort studies.

Eligibility of titles and abstracts was independently assessed by two reviewers, and disagreements were resolved by consensus with the participation of a third reviewer. Studies were considered eligible when UVC was reported as a primary risk factor for PVT. Potentially eligible articles had their full texts retrieved and analyzed. Both selection phases were performed using the Rayyan tool, and the selection process was documented in accordance with the PRISMA guidelines [34].

### Inclusion criteria

Studies were considered eligible if they met the following criteria:

• Evaluated PVT prevalence in neonates.
• Reported prevalence of PVT among neonates with umbilical venous catheterization.
• Described and analyzed risk factors associated with PVT development.
• Included ultrasound investigations for PVT detection in catheterized and non-catheterized patients (if control group available).
• Published as full-text articles with detailed methodology.
• Published in peer-reviewed academic journals.

### Exclusion criteria

The following were excluded in the first screening stage:

• Studies not addressing UVC as a primary risk factor for PVT.
• In vitro, experimental, animal, or autopsy studies.
• Case reports.
• Systematic reviews and meta-analyses.
• Conference abstracts, editorials and letters to the editor lacking methodological and/or results description.
• Articles without complete outcome and risk factor data.
• Publications without an abstract.

In the second screening stage, the following were excluded:

• Retrospective studies.
• Studies with fewer than 10 neonates.
• Studies with overlapping data from the same institution.
• Studies evaluating different devices coupled to the umbilical catheter that could interfere with catheter-related complications.
• Studies not distinguishing PVT from other hepatic complications: studies in which portal vein thrombosis was not analyzed as a distinct outcome but was instead grouped with other hepatic complications (e.g., hepatic parenchymal injury or liver abscess) were excluded from the analysis.
• Studies investigating PVT in patients with liver comorbidities.
• Articles published in journals with undefined pagination.
• Inability to access full-text articles in indexed databases after title/abstract review.

### Quality assessment

Study quality was assessed using the Newcastle–Ottawa Scale (NOS), an appropriate tool for non-randomized comparative observational studies and consistent with the cohort designs included in this review [33].

### Data analysis

Statistical analysis was performed using R software (R Foundation for Statistical Computing, Vienna, Austria). Meta-analysis of proportions and their corresponding confidence intervals was conducted using a random-effects model fitted via linear models. Pooled estimates were obtained using the “metaprop” function from the “meta” package in R. Heterogeneity was evaluated using Cochran’s Q test and Higgins and Thompson’s I² statistic. Results were summarized in forest plots, and publication bias was assessed using funnel plots and Egger’s test. To compare the outcome between groups, meta-analyses were performed to estimate the summary odds ratio with the respective confidence interval, using the “metabin” function from the “meta” package. Sample heterogeneity was assessed by Cochran’s Q test, with an adopted significance level of 5%.

For data analysis, only patients with a history of umbilical vein catheterization were included; the following variables were examined:

• (N1) = number of catheterized patients with PVT / (N2) = number without PVT.
• (GA1) = number of preterm catheterized neonates / (GA2) = number of term catheterized neonates (gestational age).
• (W1) = number of catheterized neonates with birth weight ≤ 1500 g / (W2) = number with birth weight > 1500 g.
• (T1) = catheter dwell time ≥ 7 days / (T2) = catheter dwell time < 7 days.
• (L1) = catheter tip in intrahepatic or peripheral position / (L2) = catheter tip in central position — peripheral and intrahepatic catheter tip positions are considered suboptimal for umbilical catheter placement according to the medical literature, and were therefore grouped into a single variable [7,12,14,35,36].
• Independent risk factors: neonatal sepsis, neonatal asphyxia, necrotizing enterocolitis (NEC), dehydration, respiratory distress syndrome (RDS), small for gestational age (SGA), maternal diabetes, maternal hypertension or preeclampsia, chorioamnionitis, antenatal corticosteroid use, blood transfusion or exchange transfusion through the umbilical catheter, thrombophilia/family history of thrombophilia.
• Diagnostic method: abdominal ultrasonography as an imaging modality for PVT screening.

### Ethical considerations

This systematic review and meta-analysis did not involve direct patient data collection and therefore did not require formal research ethics approval. However, all included studies were conducted in compliance with ethical standards for research involving human subjects.

## Results

An initial search of indexed databases identified 344 relevant studies on PVT associated with UVC. After duplicate removal, 166 studies were considered eligible for title and abstract screening. Of these, 116 were excluded after review and consensus among the three reviewers. From 50 potentially eligible studies, 39 were excluded after full-text review.

**Figure 1:**
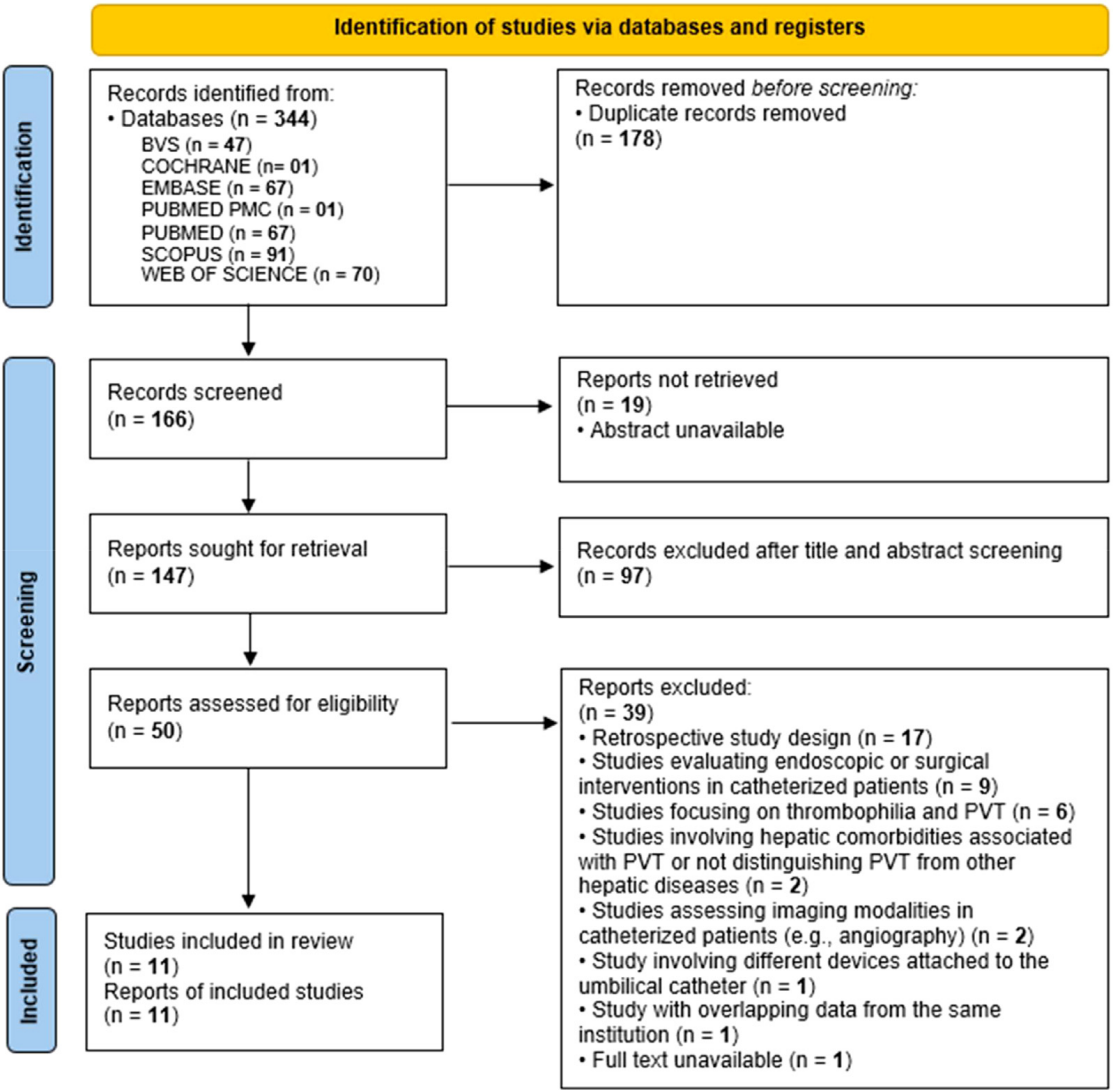
PRISMA flow diagram of the study selection process.

Ultimately, 11 studies met all inclusion criteria and were selected for data extraction and statistical analysis. Methodological quality, assessed using the Newcastle-Ottawa Scale, ranged from moderate to high across all eligible studies. Details on article selection are presented in the PRISMA flow diagram (Figure 1).

After data extraction, only risk factors reported in at least three of the 11 studies and suitable for categorization were included in the statistical analysis. Meta-analysis to obtain pooled odds ratios with confidence intervals and to assess heterogeneity using Cochran’s Q test was performed only for the groups related to catheter tip location, neonatal sepsis, and blood transfusion or exchange transfusion through the umbilical catheter. Analysis of gestational age, birth weight, and catheter dwell time was not feasible, as most studies reported these variables solely as means and standard deviations, which precluded their inclusion in the statistical models.

This meta-analysis included 11 studies comprising a total of 1086 neonates, of whom 1052 underwent UVC during the neonatal period. Sample sizes among the included studies ranged from 38 to 213 catheterized neonates. Abdominal ultrasound was performed in all 1052 patients, with 200 confirmed cases of PVT, yielding a prevalence of 19%, as summarized in Table 1, Table 2.

**Table 1:** Characteristics of catheterized patients with PVT^a^.

	N UVC	N1	GA1	GA2	W1	W2	T1	T2	L1	L2	R1	S1
**Abd El Gawad, 2021**	100	0 (0%)	0 (0%)	0 (0%)	0 (0%)	0 (0%)	-	-	0 (0%)	0 (0%)	0 (0%)	0 (0%)
**Cabannes, 2018**	104	51 (49%)	51 (100%)	0 (0%)	-	-	1 (2%)	50 (98%)	30 (58,8%)	21 (41,2%)	18 (35,3%)	0 (0%)
**Çakir, 2020**	96	13 (13,5%)	13 (100%)	0 (0%)	-	-	-	-	7 (53,8%)	6 (46,2%)	3 (23,1%)	-
**Colella, 2024**	213	57 (26,7%)	-	-	0 (0%)	57 (100%)	-	-	24 (42,1%)	33 (57,9%)	16 (28,1%)	0 (0%)
**Gharehbaghi, 2011**	164	5 (3%)	-	-	-	-	-	-	-	-	-	2 (40%)
**Guimarães, 1998**	40	0 (0%)	-	-	-	-	0 (0%)	0 (0%)	0 (0%)	-	0 (0%)	0 (0%)
**Kim, 2001**	100	43 (43%)	-	-	15 (34,9%)	28 (65,1%)	23 (53,5%)	20 (46,5%)	16 (37,2%)	27 (62,8%)	5 (11,6%)	26 (60,4%)
**Maamouri, 2016**	38	1 (2,6%)	-	-	-	-	0 (0%)	1 (100%)	-	-	0 (0%)	1 (100%)
**Sakha, 2007**	50	17 (34%)	3 (17,6%)	14 (82,4%)	0 (0%)	17 (100%)	0 (0%)	17 (100%)	-	-	11 (64,7%)	17 (100%)
**Schwartz, 1997**	100	1 (1%)	1 (100%)	0 (0%)	0 (0%)	1 (100%)	0 (0%)	1 (100%)	0 (0%)	1 (100%)	-	-
**Yadav, 1993**	47	12 (25,5%)	-	-	-	-	0 (0%)	12 (100%)	-	-	7 (58,3%)	12 (100%)
**Total:**	**1052**	**200**	**68**	**14**	**15**	**103**	**24**	**101**	**77**	**88**	**60**	**58**

a Abbreviations: UVC = umbilical venous catheter; N = total number of catheterized patients included in the study; N1 = number of catheterized patients with PVT; GA1 = preterm neonates; GA2 = term neonates; W1 = birth weight ≤ 1500 g; W2 = birth weight > 1500 g; T1 = catheter dwell time ≥ 7 days; T2 = catheter dwell time < 7 days; L1 = catheter tip in an intrahepatic or peripheral position; L2 = catheter tip in a central position; R1 = neonatal sepsis diagnosis in patients with PVT; S1 = prior blood transfusion or exchange transfusion via the umbilical catheter in neonates with PVT; **–** = data not available (NA).

**Table 2:** Characteristics of catheterized patients without PVT^a^.

	N UVC	N2	GA1	GA2	W1	W2	T1	T2	L1	L2	R1	S1
**Abd El Gawad, 2021**	100	100 (100%)	77 (77%)	23 (23%)	-	-	-	-	25 (25%)	75 (75%)	18 (18%)	4 (4%)
**Cabannes, 2018**	104	53 (51%)	53 (100%)	0 (0%)	-	-	1 (1,9%)	52 (98,1%)	19 (35,8%)	34 (64,2%)	23 (43,3%)	0 (0%)
**Çakir, 2020**	96	83 (86,4%)	83 (100%)	0 (0%)	-	-	-	-	59 (71%)	24 (29%)	22 (26,5%)	-
**Colella, 2024**	213	156 (73,2%)	-	-	0 (0%)	156 (100%)	-	-	59 (37,8%)	97 (62,2%)	27 (17,3%)	0 (0%)
**Gharehbaghi, 2011**	164	159 (96,9%)	-	-	-	-	-	-	-	-	-	12 (7,5%)
**Guimarães, 1998**	40	40 (100%)	-	-	-	-	0 (0%)	40 (100%)	40 (100%)	-	0 (0%)	40 (100%)
**Kim, 2001**	100	57 (57%)	-	-	16 (28,1%)	41 (71,9%)	6 (10,5%)	51 (89,5%)	20 (35,1%)	37 (64,9%)	4 (7%)	21 (36,8%)
**Maamouri, 2016**	38	37 (97,3%)	-	-	-	-	-	-	-	-	5 (13,5%)	12 (32,4%)
**Sakha, 2007**	50	33 (66%)	7 (21,2%)	26 (78,8%)	0 (0%)	33 (100%)	0 (0%)	33 (100%)	-	-	4 (12,1%)	33 (100%)
**Schwartz, 1997**	100	99 (99%)	-	-	-	-	-	-	-	-	-	-
**Yadav, 1993**	47	35 (74,4%)	-	-	-	-	0 (0%)	35 (100%)	-	-	4 (11,4%)	35 (100%)
**Total:**	**1052**	**852**	**220**	**49**	**16**	**230**	**7**	**211**	**222**	**267**	**107**	**157**

a Abbreviations: UVC = umbilical venous catheter; N = total number of catheterized patients included in the study; N2 = number of catheterized patients without PVT; GA1 = preterm neonates; GA2 = term neonates; W1 = birth weight ≤ 1500 g; W2 = birth weight > 1500 g; T1 = catheter dwell time ≥ 7 days; T2 = catheter dwell time < 7 days; L1 = catheter tip in an intrahepatic or peripheral position; L2 = catheter tip in a central position; R1 = neonatal sepsis diagnosis in patients without PVT; S1 = prior blood transfusion or exchange transfusion via the umbilical catheter in neonates without PVT; **–** = data not available (NA).

**Figure 2:**
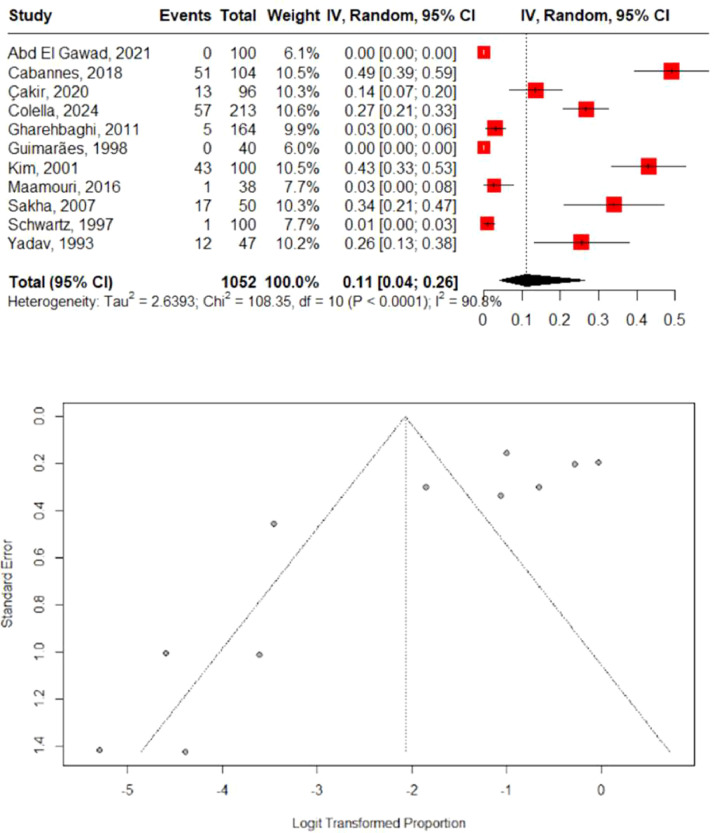
Meta-analysis of PVT prevalence among catheterized neonates and funnel plot for assessment of publication bias.

Six of the 11 studies reported a PVT prevalence above 5%. Prevalence estimates varied widely, ranging from 0% to 49%. Substantial heterogeneity was observed (I² = 90.8%; *p* < 0.0001). Assessment of publication bias using Egger’s test demonstrated significant funnel plot asymmetry (*p* = 0.01) (Figure 2).

Regarding demographic variables such as gestational age and birth weight, the presentation of these variables as mean values in most studies limited statistical assessment. Among the studies reporting precise values, preterm neonates predominated in both outcomes (83% of PVT cases and 81.2% of those without PVT), reflecting the greater need for catheterization in premature infants.

Catheter indwelling time was categorized in five studies (*n* = 312), with reported durations ranging from 120 min (Guimarães et al.) to 50 days (Maamouri et al.) [5,37]. Among these 312 patients, 125 developed PVT, including 101 in the < 7-day group and 24 in the ≥ 7-day group. For durations ≥ 7 days, the PVT rate was 77.4%, although this result was largely driven by a single study (Kim et al.), in which 23 of the 24 prolonged catheterizations resulted in PVT [12]. In contrast, patients with catheter dwell time < 7 days had a PVT prevalence of 32.4%. This subgroup was larger, as most studies reported catheter use for short procedures, such as exchange transfusion [5,[37], [38], [39]] or therapeutic hypothermia for neonatal asphyxia [11]. The 7-day cutoff may have been overly broad, and more refined stratification intervals could provide a more precise risk evaluation [40].

### Association between PVT and catheter tip location

**Figure 3:**
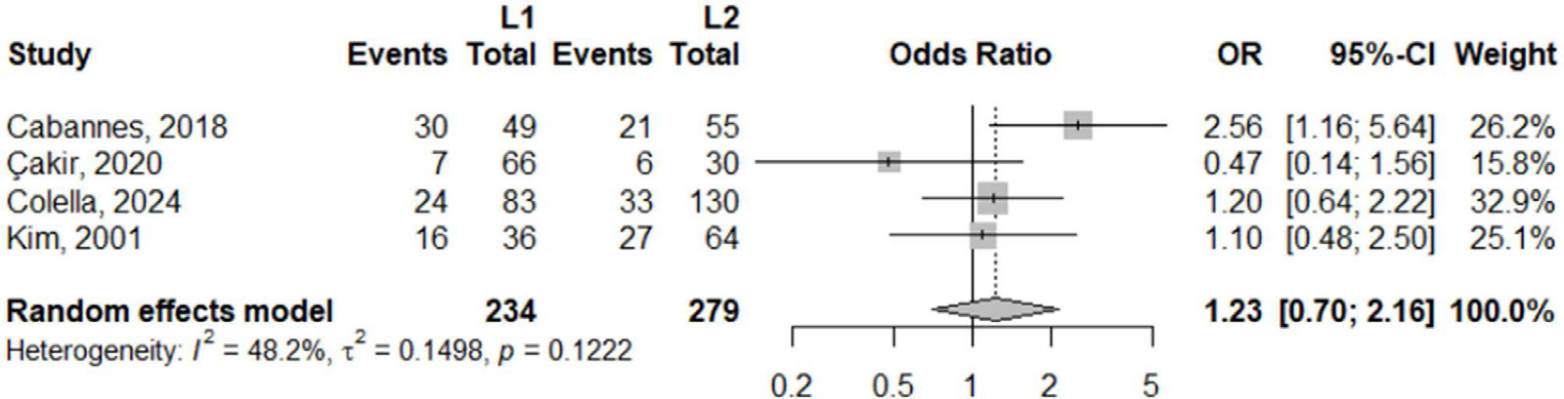
Meta-analysis of PVT according to umbilical catheter tip location (L1: intrahepatic or peripheral; L2: central).

Catheter tip location, confirmed by chest and abdominal radiography, was reported in four studies (*n* = 513). Tip positions were classified as central (adequate) or intrahepatic/peripheral (inadequate, analyzed as a single category). Among neonates with PVT, 53.3% had central catheter placement and 46.7% had intrahepatic/peripheral placement. Comparable proportions were observed among those without PVT (54.6% vs. 45.4%). No statistically significant association was found (OR = 1.23; 95% CI: 0.70–2.16; *p* = 0.4636). The sample showed moderate heterogeneity with a potential risk of bias (I² = 48.2%; *p* = 0.1222) (Figure 3).

### Association between PVT and neonatal sepsis

Neonatal sepsis, either clinically diagnosed or confirmed by positive blood cultures, was evaluated in seven studies (*n* = 648). Sepsis prevalence was 33.3% among neonates with PVT compared with 18.1% among those without PVT.

**Figure 4:**
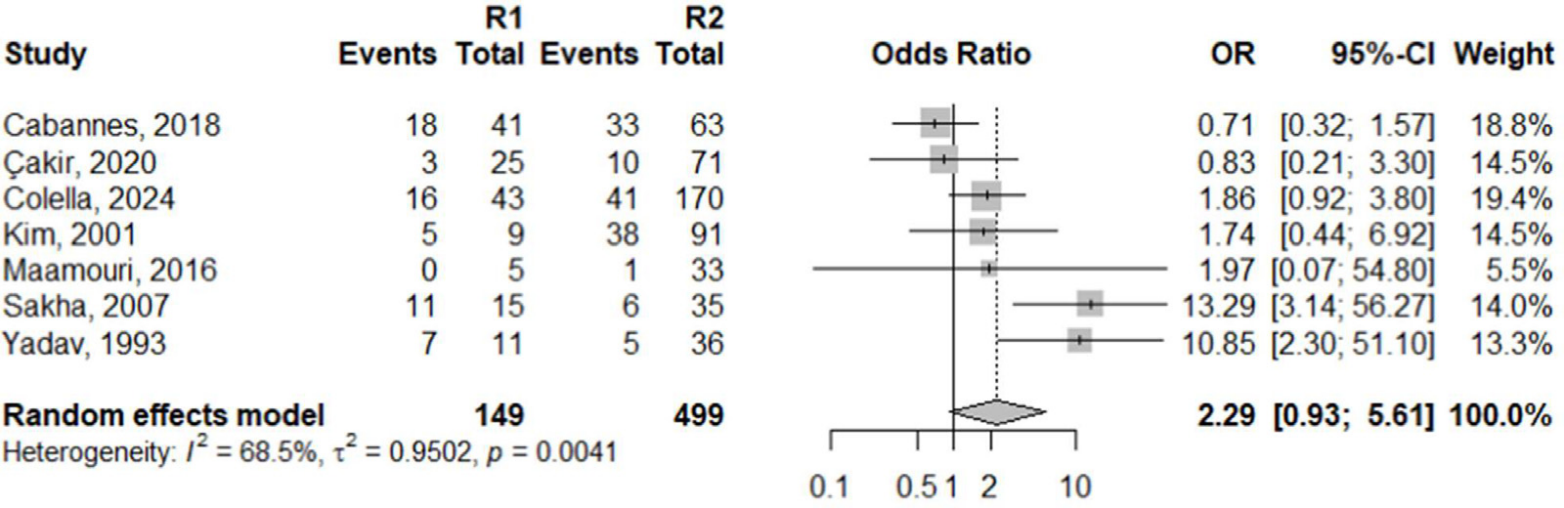
Meta-analysis of PVT according to neonatal sepsis diagnosis (R1: diagnosed neonatal sepsis; R2: no diagnosis of neonatal sepsis).

Combined analysis suggested a trend toward an increased risk of PVT in neonates with sepsis (OR = 2.29; 95% CI: 0.93–5.61), although this association did not reach statistical significance (*p* = 0.0705). Heterogeneity was high (I² = 68.5%; *p* = 0.0041) (Figure 4). Funnel plot assessment revealed no evidence of publication bias (*p* = 0.3414).

### Association between PVT and blood transfusion or exchange transfusion through the umbilical catheter

**Figure 5:**
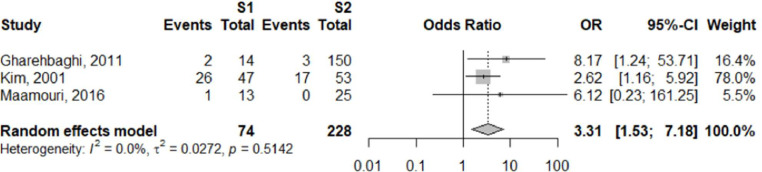
Meta-analysis of PVT according to prior blood transfusion or exchange transfusion via the umbilical catheter (S1: yes; S2: no).

Three studies (*n* = 302) evaluated the association between PVT and blood transfusion through the umbilical catheter. PVT prevalence was 27% among neonates receiving blood components via the catheter, compared with 20% in those unexposed to this risk factor. The meta-analysis demonstrated a statistically significant association (OR = 3.31; 95% CI: 1.53–7.18; *p* = 0.0024), with complete homogeneity across studies (I² = 0%; *p* = 0.5142) (Figure 5).

### Ultrasonographic findings in patients with PVT

Six studies reported ultrasound findings in neonates with PVT. The primary variables assessed were thrombus size and its anatomical location within the portal venous system. Among the 136 neonates for whom thrombus extent was documented, 58% presented partial occlusion and 42% had complete occlusion of the portal vein. Thrombus location was specified in 176 of 200 cases: 98.3% (*n* = 173) were located in the left portal vein branch, 1.7% (*n* = 3) in the main portal vein trunk, and none involved the right branch. Table 3, Table 4 present, respectively, the ultrasound screening protocols adopted in the included studies and the key imaging features of PVT.

**Table 3:** Ultrasound screening protocols and imaging findings of portal vein thrombosis (PVT) reported in the included studies^a^.

	Timing of Initial Screening	Screening Interval	Post-discharge Follow-up	Ultrasound Findings and PVT Classification
**Abd El Gawad, 2021**	At 1 month after catheter insertion	At 1, 6, and 12 months after catheterization	Follow-up for all infants up to 12 months of age	No PVT detected
**Cabannes, 2018**	On day 3 of life*	On days 3, 10, and 45 of hospitalization (or before discharge)	Follow-up at 6 months and 1 year if persistent PVT	Partial (non-occlusive) PVT; occlusive PVT
**Çakir, 2020**	Median 4 days (range 2–30) after catheter removal	Follow-up at 48–72 h if PVT on initial assessment	Follow-up until thrombus resolution⁎⁎	Partial PVT; complete or progressive PVT
**Colella, 2024**	Between days 5 and 10 of life, at least 2–3 days after catheter removal	In accordance with standardized NICU practice	Follow-up until thrombus resolution in anticoagulated patients	PVT grading (Morag classification, grades 1–3)***
**Gharehbaghi, 2011**	Within 24–48 h of catheter insertion	At 24–48 h after catheter insertion, 48–72 h after catheter removal, and weekly in patients with PVT until thrombus resolution or hospital discharge	Weekly follow-up in patients with PVT (3–6 weeks)	Partial (non-occlusive) PVT; occlusive PVT
**Guimarães, 1998**	At school age	Single US examination	No data	Left hepatic lobe hypotrophy; left portal vein not visualized
**Kim, 2001**	During catheterization, within 1 week after catheter insertion (median 2 days)	Every 2–4 days until catheter removal; for persistent PVT, additional follow-up every 2–7 days until thrombus resolution or hospital discharge	Outpatient follow-up for unresolved thrombi: median 8 days (range 2–73 days)	Partial (non-occlusive) PVT; occlusive PVT Complete resolution; reduction in size or extent; persistent PVT; progressive PVT
**Maamouri, 2016**	Before 3 years of age	Single US examination	No data	Portal vein dilation (14 mm); hepatomegaly; splenomegaly
**Sakha, 2007**	Within 1–2 weeks after catheter removal	Follow-up at 1–2-month intervals until thrombus resolution	Follow-up until thrombus resolution (2–5 months)	Non-occlusive PVT; occlusive PVT
**Schwartz, 1997**	Age 5 days–5 months (median 2 months), within 1 week prior to discharge	Single US examination	Follow-up until thrombus resolution (6 months)	Non-occlusive PVT; occlusive PVT
**Yadav, 1993**	Group A: within 4–8 weeks of birth (catheterized patients) Group B: at 1–5 years of age (children with prior catheterization) Group C: at 4–8 weeks of age (healthy infants, control group)	Group A: Follow-up at 3-month intervals until 12 months (3–6 months; 7–9 months; 10–12 months) and at 24 months of age Group B: Single US examination Group C: Single US examination	Follow-up for Group A only, up to 24 months of age	Signs of splenoportal obstruction: non-visualization of the left portal and splenic veins

⁎ In catheterized patients, US was performed during catheterization; the same timing was applied to controls.

⁎⁎ In anticoagulated patients, median treatment duration was 30 days (range 15–50), with thrombus resolution occurring 7–120 days post-treatment; in untreated patients, resolution occurred within 30–105 days.

⁎⁎⁎ PVT graded according to Morag et al. [11]: grade 1, non-occlusive PVT with normal liver parenchyma; grade 2, occlusive PVT with normal liver parenchyma; grade 3, occlusive PVT with liver parenchymal abnormalities on ultrasound.

a Abbreviations: US = Ultrasound; PVT = Portal Vein Thrombosis; NICU = Neonatal Intensive Care Unit.

**Table 4:** Clinical outcomes and follow-up data in patients with PVT.

	N PVT	O1	O2	L1	L2	L3	T	R	P
**Abd El Gawad, 2021**	0	0 (0%)	0 (0%)	0 (0%)	0 (0%)	0(0%)	0 (0%)	0 (0%)	0 (0%)
**Cabannes, 2018**	51	-	-	51 (100%)	-	-	0 (0%)	46 (90,2%)	5 (9,8%)
**Çakir, 2020**	13	8 (61,5%)	5 (38,5%)	-	-	-	11 (84,6%)	13 (100%)	0 (0%)
**Colella, 2024**	57	28 (49,1%)	29 (50,9%)	54 (94,7%)	3 (5,3%)	0 (0%)	-	-	-
**Gharehbaghi, 2011**	5	0 (0%)	5 (100%)	5 (100%)	-	-	-	3 (60%)⁎	-
**Guimarães, 1998**	0	0 (0%)	0 (0%)	0 (0%)	0 (0%)	0 (0%)	0 (0%)	0 (0%)	0 (0%)
**Kim, 2001**	43	26 (60,4%)	17 (39,6%)	43 (100%)	0 (0%)	0 (0%)	0 (0%)	20 (46,5%)⁎	16 (37,2%)⁎
**Maamouri, 2016**	1	-	-	-	-	-	-	0 (0%)	1 (100%)
**Sakha, 2007**	17	16 (94,1%)	1 (5,9%)	17 (100%)	0 (0%)	0 (0%)	0 (0%)	13 (76,4%)⁎	-
**Schwartz, 1997**	1	1 (100%)	0 (0%)	1 (100%)	0 (0%)	0 (0%)	0 (0%)	1 (100%)	0 (0%)
**Yadav, 1993**	12	-	-	-	-	-	0 (0%)	6 (50%)	6 (50%)
**Total:**	200	79	57	171	3	0	11	102	28

*Abbreviations:* N PVT = number of catheterized patients with PVT; O1 = partial portal vein occlusion; O2 = complete portal vein occlusion; L1 = thrombus location: left portal vein branch; L2 = thrombus location: main portal vein; L3 = thrombus location: right portal vein branch; T = number of patients receiving anticoagulation; R = thrombus resolution (spontaneous or post-treatment) within 1 year; P = thrombus persistence after 1 year; - = data not available (NA).

⁎ Outcome data were not available for all patients due to loss to follow-up.

## Discussion

### Analysis of risk factors identified across studies

#### Blood transfusion via umbilical venous catheters as a risk factor for PVT

This meta-analysis demonstrated a statistically significant association between blood transfusion or exchange transfusion through the umbilical catheter and the occurrence of PVT in neonates undergoing UVC (*p* = 0.0024). The heterogeneity test indicated homogeneity across studies (*p* = 0.5142), strengthening the reliability of this estimate. In particular, Kim et al. identified a significant association between PVT and the use of umbilical catheters for transfusion. In their study, this approach was used in 26 of 43 neonates with PVT (60.4%), compared with 21 of 57 neonates without PVT (36.8%) (*p* = 0.019), consistent with the findings of the present meta-analysis [12].

In the reviewed articles, umbilical catheters were used for transfusion in two clinical contexts: exchange transfusion for severe indirect (unconjugated) hyperbilirubinemia at risk for kernicterus [5,[37], [38], [39]], and packed red blood cell transfusion for multifactorial neonatal anemia [12,13,24,41]. The use of umbilical catheters for transfusion was discouraged in the studies by Colella et al. and Cabannes et al., in accordance with their institutional guidelines, and therefore was not reported in those cohorts [6,11].

Blood transfusion may affect hemostasis through multiple mechanisms, encompassing both the mechanical and biochemical properties of red blood cells. Mechanically, erythrocytes influence blood viscosity and flow dynamics; biochemically, they contribute to hemostasis through aggregation and adhesion, interactions with the vascular endothelium, cellular signaling, humoral interactions mediated by surface proteins (including blood group antigens), participation in nitric oxide metabolism, and involvement in the conversion of prothrombin to thrombin [[42], [43], [44]].

Transfused red blood cells are subject to structural and functional alterations related to storage (“storage lesion”), which may impair erythrocyte morphology, function, and intracellular communication [43]. In neonates, transfusions constitute a disproportionately large fraction of the circulating blood volume compared with older populations, and may therefore result in significant hemodynamic changes, including variations in cerebral blood flow and arterial pressure, ultimately increasing the risk of complications such as intracranial hemorrhage [44].

The increased risk of PVT following red blood cell transfusion delivered through umbilical venous catheters may be attributed to blood hyperviscosity and reduced local flow near the portal venous system [12]. These findings support the recommendation to avoid the use of umbilical catheters for transfusion in routine practice, with peripheral access preferred, except in emergency situations or when exchange transfusion is required. However, the potential confounding effect of blood transfusion itself should be considered, as it may represent an independent thrombogenic factor.

#### Neonatal sepsis as a risk factor for PVT

Neonatal sepsis was the most frequently reported risk factor across the studies. Meta-analysis showed a trend toward an increased risk of PVT in neonates with sepsis (*p* = 0.0705). However, diagnostic criteria varied considerably: Cabannes et al. defined sepsis based on positive blood cultures and/or elevated inflammatory markers in contexts of presumed infection [6], whereas Colella et al. included positive cerebrospinal fluid cultures and clinical signs requiring >72 h of antibiotic therapy [11]. Only Gharehbaghi et al. specified the pathogens involved (*Serratia* sp., *Staphylococcus aureus*, and coagulase-negative *Staphylococcus*) [13]. Few articles distinguished early-onset from late-onset sepsis, and in most studies, the primary focus was not specified. Sepsis may induce a hypercoagulable state through inflammation, blood stasis, and coagulation factor consumption, potentially explaining the higher prevalence of PVT in infected patients [4,[45], [46], [47]]. However, the lack of consensus regarding sepsis definitions contributed to clinical heterogeneity and reduced the statistical power of the analysis, particularly for this variable.

#### Umbilical venous catheter tip position as a risk factor for PVT

Meta-analysis of catheter tip location showed no increased risk of PVT for intrahepatic or peripheral versus central positions (*p* = 0.4636). Theoretically, improper placement could favor thrombosis because portal vein flow is slower than in larger vessels [14], whereas positioning closer to the right atrium should reduce thrombotic risk [12,14]. Cabannes et al. noted that even transient proximity of the catheter tip to the hepatic circulation may injure vessels during repositioning maneuvers [6]. In their study, central catheters were retained and remained available for clinical use, while intrahepatic or peripheral ones were removed within 24 h unless no alternative access was available. Similarly, Kim et al. reported routine removal of intrahepatic catheters or repositioning to a peripheral location upon radiographic confirmation [12].

Thus, this meta-analysis did not identify catheter tip misplacement as a risk factor for PVT. This finding may be related to the shorter dwell time of malpositioned catheters, which are promptly removed or replaced once malposition is detected [6,12,14]. Such early intervention likely minimizes the risk associated with improper positioning, suggesting that reduced dwell times may help mitigate PVT risk in neonates when catheter placement is suboptimal.

### Ultrasound screening protocols for PVT: comparative analysis and clinical implications

In all included studies, PVT screening relied on abdominal ultrasound, the diagnostic modality of choice given its high sensitivity (70–90%) and specificity (99%), and its ability to visualize the portal vein in up to 97% of cases [48]. Although all studies implemented serial ultrasound assessments, the timing and number of examinations differed across screening practices. Notably, no diagnosis resulted from clinical suspicion or incidental detection; all PVT cases were identified through structured screening programs, indicating that early-stage PVT is typically asymptomatic.

In some studies, ultrasound assessment was initiated only after catheter removal, with intervals ranging from 2 days to 8 weeks [11,14,24,38,39,41]. In others, screening began while the catheter was still in place, between day 2 and day 7 of catheterization, and was repeated following catheter withdrawal [6,12,13]. By comparison, Guimarães et al., Maamouri et al., and a subset of patients in the cohort reported by Yadav et al. adopted a later screening approach beyond the neonatal period, with individuals subsequently recalled for ultrasound evaluation during infancy or at school age [5,37,39].

The timing of screening initiation may influence the reported prevalence of PVT. Protocols incorporating early ultrasound assessment, still during the catheterization period, are more likely to detect thrombi before spontaneous resolution occurs [24]. Supporting this hypothesis, Gharehbaghi et al. reported a mean age of 6.39 ± 2.33 days at the time of detection (range: 3–12 days), suggesting that PVT can be identified within the first days of umbilical catheterization [13].

The high prevalence reported by Kim et al. (43%) may also be associated with early ultrasound screening. In their study, portal vein recanalization was more frequently observed in patients with non-occlusive PVT [12], providing evidence that ultrasound performed in the first few days after catheter insertion allows timely catheter removal when PVT is identified, thereby preventing thrombus progression to complete portal vein occlusion and reducing the risk of portal hypertension. Delayed initiation of screening, as highlighted by Çakir et al., may limit the ability to identify the timing of thrombus formation and lead to underestimation of its occurrence, as smaller, rapidly resolving thrombi may go undetected, contributing to lower reported prevalence rates in studies adopting this approach [24].

Commonly assessed structures included the portal vein trunk and its right and left branches, hepatic veins, splenic vein, and superior mesenteric vein, with Doppler employed to detect thrombi and/or identify evidence of flow obstruction. Yadav et al. further expanded the protocol by assessing splenic vein patency and spleen size. Although no thrombi were identified, neonates with non-visualization of the splenic vein were classified as suspected PVT and followed until the vessel could be adequately visualized [39]. The authors proposed that splenic vein non-visualization after umbilical catheterization may constitute an indirect marker of PVT.

The most divergent ultrasound criteria were noted across the Guimarães et al. and Kim et al. studies. Guimarães et al. described two neonates with absent visualization of the left portal branch (one of whom also exhibited reduced left-lobe size), findings they interpreted as normal anatomical variants, thereby yielding a PVT prevalence of 0% in their cohort [37]. Meanwhile, Kim et al. considered comparable findings to represent late sequelae of PVT [12].

Resolution of PVT was frequently observed in the included studies. Among patients who were followed, resolution occurred in 78.3% of cases, either spontaneously or following medical intervention, within a period of up to one year. However, 21.7% of patients exhibited persistent thrombosis after this interval. Notably, most cases resolved even in the absence of therapeutic intervention, as a conservative management approach was adopted across most series.

Although some studies have reported promising results with anticoagulant use, evidence supporting most recommendations for antithrombotic therapy in neonates and children remains limited [49,50], and no consensus has been reached regarding its effectiveness in reducing time to thrombus resolution or decreasing the risk of progression to portal hypertension [24]. Among conservative strategies, Cabannes et al. reported favorable outcomes in >90% of cases analyzed [6], while Kim et al. identified a significant correlation (*p* = 0.024) between initial thrombus size and the likelihood of spontaneous resolution, suggesting that occlusive thrombi are associated with higher rates of persistence, and require closer surveillance for portal hypertension–related complications [4,11,12,24]. These findings, together with the uncertainty surrounding the efficacy and safety of anticoagulant therapy in neonates, underscore the importance of early detection of PVT by abdominal ultrasound, regardless of the therapeutic approach.

Considering the association between UVC and PVT, particularly in the presence of risk factors such as neonatal sepsis and catheter-mediated blood transfusion, early detection is essential for risk stratification and clinical decision-making. Longitudinal follow-up of these patients permits continuous monitoring of complications related to portal hypertension, with therapeutic management primarily aimed at preventing clinical manifestations, including hypersplenism, gastroesophageal variceal bleeding, portal hypertensive gastropathy, portal biliopathy, and hepatopulmonary syndrome [51]. The primary goal in patients with OEHVP is to prevent gastrointestinal bleeding [21], guiding primary and secondary prophylaxis for variceal bleeding, as well as surgical intervention when indicated [21,[51], [52], [53]]. Emerging evidence also supports early surgical treatment in selected patients, even in the absence of severe complications [53].

The findings of this study highlight the importance of early ultrasound screening in neonates undergoing UVC to identify those at increased risk of progression to portal hypertension, and to enable timely detection of complications amenable to intervention, such as the development of esophageal varices and the need for primary prophylaxis of UGIB. In addition, caution is warranted when using umbilical venous catheters for blood transfusion, especially if alternative approaches are available. Collectively, these strategies may help reduce morbidity and improve clinical outcomes in this population.

### Study limitations

This meta-analysis, despite its contributions, is not without limitations. These include high heterogeneity across studies, which restricted the statistical analysis of risk factors apart from transfusion; the absence of non-catheterized control groups in most cohorts; the lack of standardized screening protocols; divergent ultrasound diagnostic criteria (for example, treating non-visualization of the left portal branch as diagnostic of PVT in some studies but not in others); and potential publication bias, as suggested by the funnel plot and Egger’s test.

The high heterogeneity (I² = 90.8%; *p* < 0.0001) may reflect true clinical and methodological variability across studies, likely due to institutional differences. Patient populations were also heterogeneous, with comorbidities that differed considerably and were seldom reported consistently between cohorts. Moreover, common neonatal risk factors, such as neonatal asphyxia, NEC, and RDS, were reported in fewer than three studies, preventing their inclusion in the quantitative analysis.

The absence of non-catheterized control groups in 9 of the 11 studies limited the assessment of UVC as an independent risk factor for PVT. Nonetheless, this limitation is partially justified by the clinical imperative to perform the procedure in circumstances such as extreme prematurity, hemodynamic instability at birth, indications for exchange transfusion, or the lack of a feasible alternative for venous access when vascular access is needed [36].

Reported PVT prevalence ranged from 0% to 49%, consistent with the findings of Lopes et al. [15]. Although heterogeneous, more than half of the included studies reported prevalence rates exceeding 5%, indicating a substantial likelihood of PVT among catheterized neonates and reinforcing the rationale for systematic screening with serial abdominal ultrasound in this population.

## Conclusions

The prevalence of PVT identified in this systematic review was 19%, with individual studies reporting rates ranging from 0% to 49%. The majority of studies reported a prevalence greater than 5%. Substantial heterogeneity was observed, with 90.8% of the total variability attributable to genuine differences among the included cohorts.

The meta-analysis of risk factors demonstrated a statistically significant association between blood transfusion or exchange transfusion performed through the umbilical catheter and the development of PVT. Furthermore, the analysis showed a trend toward an increased risk of PVT in patients with a history of neonatal sepsis.

Based on these findings, the routine implementation of systematic PVT screening protocols is recommended for all newborns who have undergone UVC during the neonatal period.

## Funding

The authors received no financial support for the research or authorship of this article.

## Data availability

All data analyzed in this study are derived from previously published sources and are included in the article and its supplementary material.

## Declaration of generative AI and AI-assisted technologies in the manuscript preparation process

During the preparation of this work the authors used Chat-GPT (OpenAI) solely in order to assist with language editing and translation into English, without generating any original scientific content. After using this tool, the authors reviewed and edited the content as needed and takes full responsibility for the content of the published article.

## Conflicts of interest

The authors declare no conflicts of interest.
